# Impact of Phenol-Enriched Olive Oils on Serum Metabonome and Its Relationship with Cardiometabolic Parameters: A Randomized, Double-Blind, Cross-Over, Controlled Trial

**DOI:** 10.3390/antiox11101964

**Published:** 2022-09-30

**Authors:** Marta Farràs, Jonathan Richard Swann, Ian Rowland, Laura Rubió, Isaac Subirana, Úrsula Catalán, Maria José Motilva, Rosa Solà, Maria Isabel Covas, Francisco Blanco-Vaca, Montserrat Fitó, Jordi Mayneris-Perxachs

**Affiliations:** 1 Unit of Nutrition and Cancer, Epidemiology Research Program, Catalan Institute of Oncology (ICO), Bellvitge Biomedical Research Institute (IDIBELL), L’Hospitalet de Llobregat, 08908 Barcelona, Spain; 2 Division of Metabolism, Digestion, and Reproduction, Faculty of Medicine, Imperial College, London SW7 2BX, UK; 3 Institut d’Investigacions Biomèdiques (IIB) Sant Pau, C/Sant Quintí 77, 08041 Barcelona, Spain; 4 School of Human Development and Health, Faculty of Medicine, University of Southampton, Southampton SO17 1BJ, UK; 5 Hugh Sinclair Human Nutrition Group, University of Reading, Reading RG6 6DH, UK; 6 Antioxidants Research Group, Food Technology Department, AGROTECNIO-CERCA Center, University of Lleida, Avda/Alcalde Rovira Roure 191, 25198 Lleida, Spain; 7 CIBER de Enfermedades Cardiovasculares (CIBERCV), C/Monforte de Lemos 3-5, 28029 Madrid, Spain; 8 Cardiovascular Epidemiology Group, IMIM (Hospital del Mar Medical Research Institute), C/Doctor Aiguader 88, 08003 Barcelona, Spain; 9 Functional Nutrition, Oxidation, and Cardiovascular Diseases Group (NFOC-Salut), Facultat de Medicina i Ciències de la Salut, Hospital Universitari Sant Joan, Universitat Rovira i Virgili, C/Sant Llorenç 21, 43201 Reus, Spain; 10 NuProas Handesbolag (NUPROAS HB), 13141 Nacka, Sweden; 11 CIBER de Diabetes y Enfermedades Metabólicas Asociadas (CIBERDEM), C/Monforte de Lemos 3-5, 28029 Madrid, Spain; 12 Servei de Bioquímica, Hospital Santa Creu i Sant Pau—IIB Sant Pau, 08025 Barcelona, Spain; 13 Departament de Bioquímica i Biologia Molecular, Universitat Autònoma Barcelona, 08193 Barcelona, Spain; 14 CIBER Fisiopatología de la Obesidad y Nutrición (CIBERobn), Instituto de Salud Carlos III, 28029 Madrid, Spain; 15 Cardiovascular Risk and Nutrition Research Group, Regicor Study Group, IMIM (Hospital del Mar Medical Research Institute), Doctor Aiguader 88, 08003 Barcelona, Spain; 16 Department of Diabetes, Endocrinology and Nutrition, Dr. Josep Trueta University Hospital, 17190 Girona, Spain; 17 Nutrition, Eumetabolism and Health Group, Girona Biomedical Research Institute (IdibGi), 17190 Girona, Spain

**Keywords:** functional olive oil, phenolic compounds, metabonomics, cardiovascular diseases

## Abstract

Phenol-rich foods consumption such as virgin olive oil (VOO) has been shown to have beneficial effects on cardiovascular diseases. The broader biochemical impact of VOO and phenol-enriched OOs remains, however, unclear. A randomized, double-blind, cross-over, controlled trial was performed with thirty-three hypercholesterolemic individuals who ingested for 3-weeks (25 mL/day): (1) an OO enriched with its own olive oil phenolic compounds (PCs) (500 ppm; FOO); (2) an OO enriched with its own olive oil PCs (250 ppm) plus thyme PCs (250 ppm; FOOT); and (3) a VOO with low phenolic content (80 ppm). Serum lipid and glycemic profiles, serum ^1^H-NMR spectroscopy-based metabolomics, endothelial function, blood pressure, and cardiovascular risk were measured. We combined OPLS-DA with machine learning modelling to identify metabolites discrimination of the treatment groups. Both phenol-enriched OO interventions decreased the levels of glutamine, creatinine, creatine, dimethylamine, and histidine in comparison to VOO one. In addition, FOOT decreased the plasma levels of glycine and DMSO2 compared to VOO, while FOO decreased the circulating alanine concentrations but increased the plasma levels of acetone and 3-HB compared to VOO. Based on these findings, phenol-enriched OOs were shown to result in a favorable shift in the circulating metabolic phenotype, inducing a reduction in metabolites associated with cardiometabolic diseases.

## 1. Introduction

Virgin olive oil (VOO) phenolic compounds (PCs) have been reported to possess antioxidant and anti-inflammatory properties, and exert chemoprotective effects in experimental studies [1,2]. Moreover, PCs found in VOO produce beneficial changes in the serum lipid profile and haemostasis, reduce blood pressure, and have anti-thrombotic and anti-inflammatory activity in humans [3,4,5]. Data from the EUROLIVE study demonstrated an increase of HDL cholesterol (HDL-C), and a decrease in in vivo lipid oxidative damage, in an OOPC dose-dependent manner in healthy humans [6]. Furthermore, an enhancement in HDL function with virgin olive oil (VOO) in healthy humans has been reported [7]. In addition, VOO PCs have been reported to exert protection against risk factors for coronary heart disease, particularly in individuals with high oxidative stress [1,2].

Phenol-enriched foods can display a dual action since antioxidants may revert to pro-oxidant actors [8,9,10]. Functional foods with complementary PCs could therefore have their beneficial effects enhanced. In this regard, enriching OO with PCs, without increasing the fat content, could be an effective method to raise the intake of PCs and increase their potential to improve health. Our group have previously demonstrated that phenol-enriched OO improved endothelial function in pre-/hypertensive subjects [11], and enhanced endothelial and HDL functions in hypercholesterolemic individuals [12,13]. In this regard, phenol-enriched OO also increased the expression of cholesterol efflux-related genes in two transcriptomic sub-studies [14,15].

Nutritional metabolomics, or nutrimetabolomics, is being increasingly employed to study molecular interactions between diet and the global metabolic system. It allows the study of metabolic responses to dietary modulations, and permits the inter-individual variation in responses to the intake of specific nutrients and diets to be established. In addition, the metabolic phenotype contains signals derived from dietary inputs, as well as information related to the metabolic activity of the intestinal microbiota and its interactions with the diet and host. We have previously described interactions between phenol-enriched OOs and the human microbiota [16].

Food enriched with complementary antioxidants, according to their structure/activity relationship, may be a possibility to achieve healthy effects avoiding thes harmful ones. The aim of this study was to measure the serum metabolic phenotypes of hypercholesterolemic patients, and characterize the biochemical modulations induced by the intake of functional OOs enriched with their own PCs or with their own PCs plus complementary ones from thyme.

## 2. Materials and Methods

### 2.1. Phenol-Enriched Olive Oil Preparation and Characteristics

A VOO with a low-phenolic content (80 ppm) was used as a control condition and as a matrix of enrichment to prepare two functional OOs (FOOs; 500 ppm). The first was a functional VOO (FOO; 500 ppm) enriched with its own PCs by the addition of a phenolic extract obtained from freeze-dried olive cake. The phenolic profile of the olive cake extract was comparable to the control VOO, as they were obtained from the same olive variety (Arbequina cv) and the same olive-growing area. The second FOO was enriched with its own PCs and complemented with thyme PCs using a phenolic extract obtained from a mixture of freeze-dried olive cake and dried thyme (*Thymus vulgaris*) (FOOT; 500 ppm). The FOOT thus contained 50% OOPCs (some of them were naturally present in VOO and some of them came from the added olive cake extract) and 50% thyme PCs, which have been shown to possess a range of anti-inflammatory and anti-oxidative properties [17]. For the wash-out period a common OO was used. The procedure to obtain the phenolic extracts and the phenol-enriched OO has been published elsewhere [18]. Briefly, the phenolic extracts were obtained using and accelerated solvent extractor as previously optimized [18,19] obtaining an average of 2 g (1.96 ± 0.16 g) of freeze-dried phenolic extract from 10 g of raw material (freeze-dried olive cake and dried thyme). Once the phenolic extracts were obtained, they were used to prepare phenol-enriched OOs in the ratio of 2.5 g of extract/100 g of VOO with low phenolic content using a dispersion method with water (2%) until complete homogenization. The phenolic identification and quantification of the control VOO and the phenol-enriched OOs were analyzed by HPLC coupled with tandem mass spectrometry (MS/MS) as previously described [18]. As previously reported, the OOs only differed in their phenolic content, the fat and micronutrient compositions were the same (Supplementary Table S1).

### 2.2. Study Design

The VOO and HDL Functionality (VOHF) study was a randomized, double-blind, crossover, controlled trial including 33 volunteers with cholesterol levels higher than recommended (total-cholesterol > 200 mg/dL) (19 men/14 women), aged 35 to 80. Exclusion criteria were body mass index (BMI) > 35 Kg/m^2^, smokers, athletes with high physical activity (PA) (>3000 Kcal/day), diabetes, multiple allergies, intestinal or any other diseases/conditions that would worsen adherence to the measurements or treatments. The study was conducted at IMIM-Hospital del Mar Medical Research Institute (Barcelona, Spain) from April 2012 to September 2012. Subjects were allocated, by generating random numbers, to one of 3 sequences of administration of 25 mL/day of raw: (a) VOO; (b) functional OO enriched with its own PC; and (c) functional OO enriched with its own PC plus complementary phenols from thyme. The statistician was who generated the random allocation sequence while the researcher was who enrolled participants and the doctor who assigned participants to interventions according to the random sequence. Because all participants received each one of the three OOs, restrictions such as blocking were not necessary. The flow-chart of the study was previously described by Pedret et al. [20]. The intervention periods lasted 3 weeks with an ingestion of 25 mL/day raw OO distributed among meals and preceded by 2-week wash-out periods. For the wash-out period, a commercial common OO (blend of refined and a small percentage of VOO) kindly provided by Borges Mediterranean Group was used.

To avoid an excessive intake of antioxidants and PCs during the clinical trial period, participants were advised to limit the consumption of polyphenol-rich foods (such as vegetables, fruit, coffee, and olives). To register the amount of OO consumed, the volunteers had to return the OO containers to the center after each OO intervention. Participants with less than 80% of treatment adherence (≥5 full OO containers returned) were considered non-compliant subjects for this treatment. PA was evaluated by a questionnaire at baseline and cessation of the study. The trial was conducted in accordance with the Helsinki Declaration and the Good Clinical Practice for Trials on Medical Products in the European Community. All participants provided written informed consent, and the local institutional ethics committees approved the protocol (CEIC-IMAS 2009/3347/I). The protocol was registered with the International Standard Randomized Controlled Trial register (www.controlled-trials.com; ISRCTN77500181) and followed CONSORT-guidelines (Supplementary Table S2).

### 2.3. Sample Size and Power Analysis

A sample size of 30 individuals allows at least 80% power to detect a statistically significant difference among groups of 3 mg/dL of HDL cholesterol, assuming a dropout rate of 15% and a Type I error of 0.05 (2-sided).

### 2.4. Anthropometric and Cardiovascular Clinical Measurements

Blood pressure (BP), BMI, endothelial function (determined by ischemic reactive hyperaemia (IRH) as previously described [13]), and cardiovascular risk (assessed with the calibrated Framingham function [21]) were measured before and after each intervention.

### 2.5. Systemic Biomarkers

Blood samples were collected following fasting for at least 10 h. Plasma samples were obtained by centrifugation of EDTA whole blood directly after being drawn and were preserved at −80 °C until use. Plasma glucose, total-cholesterol (TC), and triglyceride (TG) levels were measured using standard enzymatic automated methods and apolipoprotein A1 (ApoA1) and apolipoprotein B-100 (APOB100) by immunoturbidimetry in a PENTRA-400 autoanalyzer (ABX-Horiba Diagnostics, Montpellier, France). HDL-C was measured using an accelerator selective detergent method (ABX-Horiba Diagnostics, Montpellier, France). LDL-C was calculated by the Friedewald equation whenever TGs were inferior to 300 mg/dL.

### 2.6. Sample Preparation and ^1^H-NMR Spectroscopy

Serum samples (200 μL) were combined with 400 μL of saline solution (100% D2O), mixed by vortexing, and centrifugated at 13,000× *g* for 10 min. The supernatant (550 μL) was transferred to a 5 mm internal diameter NMR tube. The metabolic profiles of the serum samples were then characterized by ^1^H-NMR spectroscopy using a 500 MHz Bruker NMR spectrometer operating at 310 K. For each serum sample a water-suppressed Carr-Purcell-Meiboom-Gill (CPMG) spin-echo spectrum was acquired using 8 dummy scans followed by 128 scans collected into 64K data points.

### 2.7. NMR Data Processing

^1^H-NMR spectra were manually corrected for phase and baseline distortions. Chemical shifts in the spectra were referenced to the anomeric proton of α-glucose at 5.223 ppm. Spectra were digitized using an in-house Matlab (version R2009b, The Mathworks, Inc.; Natwick, MA, USA) script. Resonances arising from imperfect water saturation were removed to minimize distortions to the spectral baseline.

### 2.8. NMR Data Analysis

#### 2.8.1. Multilevel-OPLS-DA Analyses

Metabolomics data from human studies are characterized by large variations between the subjects. Therefore, subtle treatment effects can be easily overlooked due to the considerable variation among participants due to age, disease state, genetics and the like. In addition, the impact of the treatment effect may differ between the subjects. Cross-over designed experiments are particularly useful to tackle these problems, since each subject in the study act as their own controls, making the data paired. A specific limitation of using typical techniques for metabolomics studies such as PCA, PLS or OPLS in a crossover experiments is that the net treatment effect is not separated from the biological variation between the subjects. Therefore, to identify metabolic differences between treatment groups we used a multilevel orthogonal partial least squares discriminant analysis (M-OPLS-DA) modelling [22,23]. It combines the variation splitting property of multilevel simultaneous component analysis (MSCA) with the analysis of the within subjects variation with an OPLS-DA method.

MSCA decomposes the data into an offset term, a between-subjects part and a within-subjects part [24,25]. Decomposition of the variation terms is performed in two consecutive centering steps. The first steps is applied on the entire metabolomic data set X and results in a offset term and a mean-centered data block:

$$\begin{array}{ll} X & =1_{L}x_{m}^{T}+X_{c}\\ & =X_{m}+X_{c} \end{array}$$

where *L* is the total number of spectra, X_c_ contains the mean-centered data, 1*_L_* contains ones, and $x_{m}^{T}$ contains the mean values for each column in X. The matrix X_m_ is defined as the offset term.

Then, a second mean-centering is performed per subject *i* over the *K* interventions:

$$X_{ci}=1_{K}x_{bi}^{T}+X_{wi}$$

where X_c*i*_ contains the mean-centered data for subject *i*, 1_K_ contains ones, $x_{bi}^{T}$ contains the mean for each subject *i*, and X_w*i*_ contains the mean-centered data per subject *i.* Concatenating the matrices for each individual, we can write the mean-centered data matrix as:

$$X_{c}=X_{b}+X_{w}$$

where X_b_ contains the between-subjects variation and X_w_ contains the within-subjects variation. Therefore, the metabolomic data matrix can be written as three terms containing the offset, the between-subjects variation (biological effect), and the within-subjects variation (treatment effect):

$$X=X_{m}+X_{b}+X_{w}$$

Variation splitting was performed using Matlab with the routines available from van Velzen et al. [22]: http://www.bdagroup.nl/content/Downloads/software/software.php (accessed on 1 June 2018).

After variation splitting, OPLS-DA is performed on the within-subject data to find systematic differences among the treatment groups. The within-subject variation contains both variation that is equal for all subjects as well as variation different between subjects. An OPLS-DA on the within-subjects matrix is used to focus on the similarity in the treatment effect between subjects. The OPLS-DA was introduced as modification of the PLS-DA method that incorporates orthogonal signal correction (OSC) filters to discriminate between two or more groups using multivariate data [26,27]. PLS is a multivariate regression method based on projections that models the relationship between the metabolic data set X and the response variable Y, but also models the structure of X and Y:

$$X=TP^{\prime }+E\;Y={\mathrm{UC}}^{\prime }+F$$

where T and U are the scores matrices, P and C are the loadings matrices, and E and F are the matrices of residuals of X and Y, respectively. Conversely, OPLS separates the variation in T derived from the PLS in two blocks of variation, predictive (T_p_) and orthogonal (T_o_) variation:

$$X=T_{p}{P^{\prime }}_{p}+T_{o}{P^{\prime }}_{o}+E\;Y={\mathrm{UC}}_{\prime }+F$$

The predictive block contains the correlated variation between X and Y, whereas the orthogonal block contains the uncorrelated variation between X and Y. The advantage of OPLS compared to PLS is that a single component (the predictive component) is used as a predictor of the response Y, while the other components describe the other variation that is orthogonal to Y. Therefore, the main advantage of OPLS lies on enhancing model interpretation by forcing all Y-related information into a single component. However, it is worth noting that in terms of predictive power, both PLS and OPLS give similar results [26].

OPLS-DA Loading coefficient plots were generated by back-scaling transformation where covariance is plotted between the Y-response matrix and the signal intensity of the metabolites in the NMR data (X). These plots are coloured based on the correlation coefficient (r^2^) between each metabolite and the Y-response variable, with red indicating strong significance and blue indicating weak significance. The predictive performance (Q^2^Y) of the model was calculated using a 7-fold cross-validation approach and model validity was established by permutation testing (1000 permutations).

#### 2.8.2. Machine Learning Analyses

Metabolomics datasets are often high-dimensional with non-linear and complex interactions among metabolites. Therefore, we further analysed the metabolomics data using machine learning (ML) models based on trees: random forest (RF). Unlike classical linear model-based statistical methods, RF are fully non-parametric model-free methods that capture complex interaction dependency patterns within predictor features affecting the phenotype. In addition, they provide several variable importance measures (VIMs) that can be used to identify relevant features. RF is an ensemble machine learning method based on “growing” hundreds or thousands of decision trees that uses the average output of all the trees to predict the **Y**-response [28]. Randomness in the trees is introduced by two elements. First, each tree is built from a random subset sample (bootstrap sample), with replacement, of the original data. Second, at each split in the tree building process, only a random subset of predictor features (mtry) is considered from all candidate predictors. Among these variables, the one providing the best split based on a specific criterion is selected. This has the effect of making a forest with a large number of uncorrelated trees. Then, although each individual tree may perform poorly when predicting the outcome and different trees can give different results, the ensemble of trees have better predictive capability.

The advantage of the RF is that the observations not used for the construction of a specific tree (termed out-of-bag (OOB) observations) may be used to estimate the VIM. In particular, we used the mean minimal depth, which is calculated based on the position of the features in the decision tree. Thus, unlike other VIM such as the permutation variable importance, it is only based on the structure of the forest and independent of prediction errors [29]. The RF models and the minimal depth distribution were calculated using the “ranger” and “randomForestExplainer” R packages.

However, VIMs do not provide the sign of the association with the response variable. Therefore, to facilitate model interpretation, the contribution and effect of each selected feature (i.e., metabolite) in predicting the treatment group was determined by the exact computation of SHapley Additive exPlanations (SHAP) scores by leveraging the internal structure of RF models [30]. The exact computation of SHAP values guarantees that explanations are always consistent and locally accurate. SHAP values determine the importance of a specific value in a specific feature by comparing the model prediction with and without the feature for each individual. Therefore, the same feature with a specific value may have different SHAP valued for different individuals depending on the interactions with other features of that individual. The SHAP scores were calculated and plotted using the R packages “treeshap” and “SHAPforXGBoost”, respectively.

A drawback of VIMs in RF is that they are not directly related to the statistical significance. Therefore, we further applied an all-relevant ML feature selection strategy based on applying RF iteratively as implemented in the Boruta algorithm [31]. The Boruta approach has been recently proposed as one of the best-performing RF-based variable selection methods for high-dimensional omics datasets [32]. It performs variables selection in four steps: (a) Randomization: by creating a duplicate copy of the original features randomly permutate across the observations (called shadow features); (b) Model building: build a RF with the extended data set (original + shadow features) to compute the normalized permutation VIM Z-scores for each feature; (c) Statistical testing: find those relevant features with a VIM higher than the shadow feature with the maximum VIM (MZSF) using a Bonferroni corrected two-tailed binomial test (P_Bonferroni_). Predictor features with significantly higher, significantly lower, or non-significantly different VIM Z-scores than expected at random compared to the MZSF are deemed important (selected), unimportant (rejected), or tentative, respectively; and (d) Iteration: Unimportant and shadow features are removed and the previous steps are repeated until the status of all features is decided or a predefined number of iterations has been performed. All RF models were calculated using 5000 trees and a number of features (ntree) randomly sampled at each split given by the rounded down square root of the number of features (the mtry recommended for classification problems). In addition, in the Boruta algorithm we used 500 iterations and a confidence level cut-off of 0.005 for the Bonferroni adjusted *p* values.

### 2.9. Statistical Analyses

Normality of continuous variables was assessed by normal probability plots. Non-normally distributed variables were log transformed when necessary. Non-compliant volunteers were excluded from the analysis. To compare means (for normal distributed variables) or medians (for non-normal distributed variables) among groups, ANOVA and Kruskal-Wallis test were performed, respectively. The χ2 and exact F-test, as appropriate, were used to compare proportions. A general linear model for repeated measurements was employed to assess the comparisons of changes in intra- (pre-treatment vs. post-treatment) and inter-interventions (FOOT vs. FOO vs. VOO). A value of *p*-value < 0.05 was considered significant. Spearman correlations were performed and a *p* < 0.002 was considered significant due to the assessment of 25 variables (Bonferroni *p*-value). A PLS model was also used to represent the associations between metabolites and clinical cardiovascular measures. Statistical analyses were performed by SPSS 13.0 software (IBM Corp, New York, USA) and Matlab (version R2009b, The Mathworks, Inc.; Natwick, MA, USA).

## 3. Results

### 3.1. General Characteristics, Anthropometric Measurements, and Cardiometabolic Parameters

Participants’ baseline characteristics are shown in Table 1. Order of intervention was not found to induce any significant effects in any of the variables measured. No changes in daily energy expenditure in leisure-time PA were observed from the beginning to the end of the study. No changes were observed in blood pressure, BMI, and cardiovascular risk throughout the study. IRH improved after FOOT intervention as previously described [13]. No changes were observed in lipid profile and glucose throughout the study. The three OO interventions were well tolerated by all volunteers who did not reported adverse events.

### 3.2. Serum Metabolic Phenotyping

**Figure 1:**
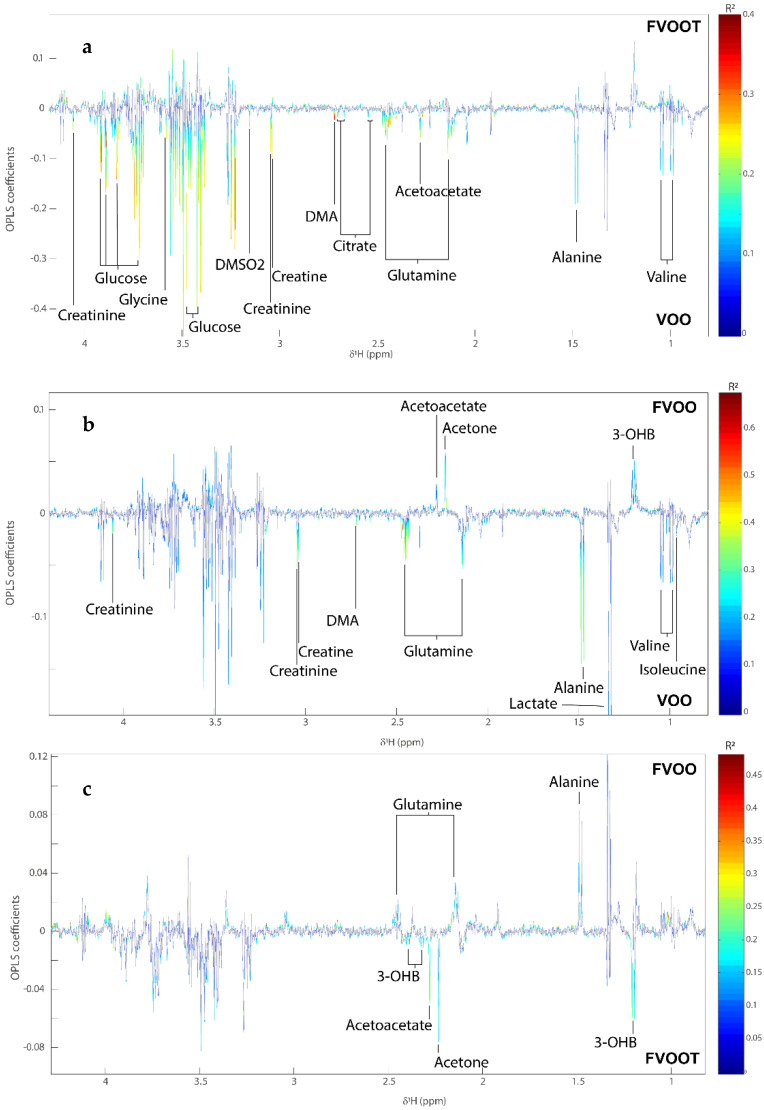
MSCA models comparing the serum metabolic profiles from study participants. MSCA coefficient plots comparing the metabolic profiles at (**a**) comparing the metabolic responses to FOOT and VOO intakes, (**b**) comparing the metabolic responses to FOO and VOO intakes, and (**c**) comparing the metabolic responses to FOOT and FOO intakes. DMA, dimethylamine; DMSO2, dimethylsufone; FOO, functional OO enriched with its own phenolic compounds; FOOT, functional OO enriched with its own phenolic compounds plus additional complementary one from thyme; VOO, virgin olive oil; 3-OHB, 3-hydroxybutyrate.

A significant MSCA model (Q^2^Y = 0.86; *p* < 0.001) was obtained comparing the serum metabolic phenotypes of individuals after receiving the VOO and FOOT interventions (Figure 1a). Compared to VOO, FOOT ingestion resulted in lower circulating amounts of valine, alanine, glutamine, histidine, and glycine, and decreased levels of citrate, creatine, creatinine, glucose, dimethylsulfone, acetoacetate, and dimethylamine (DMA) in serum. The model comparing the serum metabolic profiles after FOO and VOO intakes was also significant (Q^2^Y = 0.97, *p* < 0.001) (Figure 1b). Participants supplemented with FOO had higher amounts of 3-hydroxybutyrate (3-HB), acetate, and acetone in serum. In a similar manner to FOOT, FOO reduced levels of valine, alanine, glutamine, histidine, creatine, creatinine, and DMA in serum compared to VOO. In addition, FOO decreased lactate and isoleucine versus VOO. A significant model was also obtained comparing the serum profiles after FOOT and FOO intakes (Q^2^Y = 0.97; *p* < 0.001) (Figure 1c). Serum collected after FOO intake contained higher amounts of acetone, acetoacetate, and 3-HB and lower amounts of glutamine and alanine compared to that after FOOT intake.

**Figure 2:**
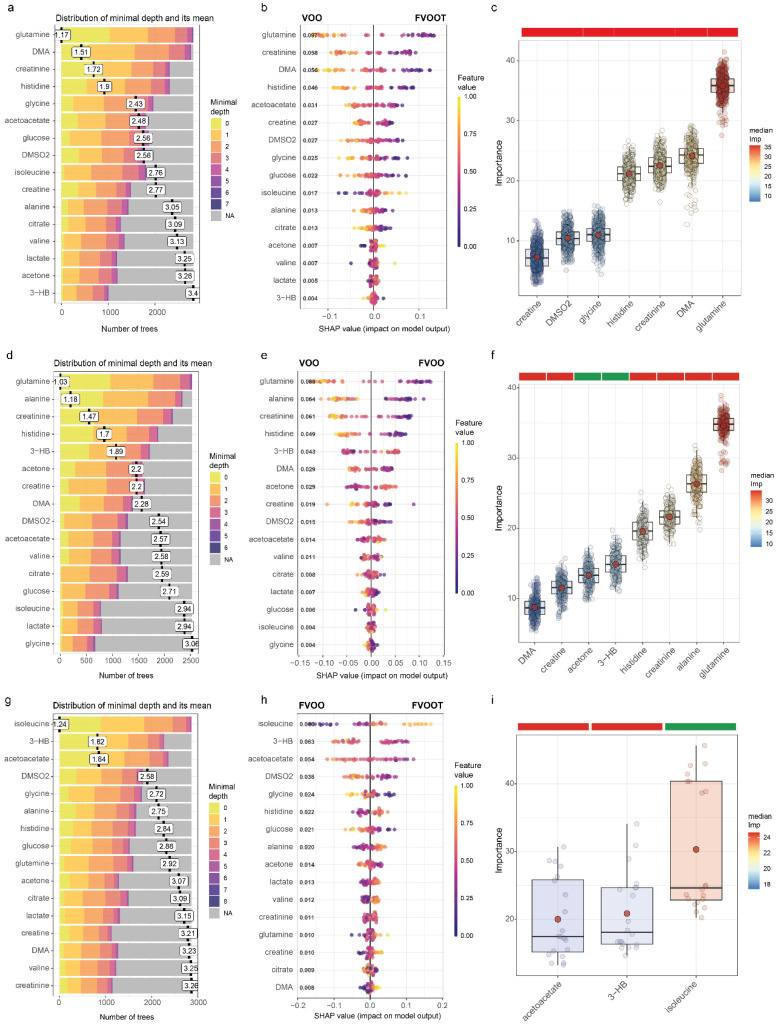
Machine learning results comparing the metabolites from study participants. (**a**–**c**) Distribution of features’ (metabolites) mean minimal depth, SHAP summary plot, and features selected by the Boruta algorithm for the comparison FOOT vs. VOO, (**d**–**f**) the comparison FOO vs. VOO, and (**g**–**i**) the comparison FOOT vs. FOO. The minimal depth distribution plot shows the features with their distribution of minimal depth and its mean, where the importance of a feature increases with decreasing mean values. In the SHAP summary plot, each dot represents and individual sample. The *X*-axis represents the SHAP value: the impact of a specific feature (metabolite) on the treatment group prediction of a specific individual. Features are sorted in decreasing order based on their overall importance for final prediction (average SHAP values shown in bold). Colours represent the values of the metabolites normalized concentrations, ranging from yellow (high concentrations of the specific metabolite) to purple (low concentrations of the specific lipid). The Boruta results are shown as boxplots of Variable Importance Measure (VIM) for each selected relevant feature. In the boxplots, the red dot represents the mean and the colour bar above each plot indicates the sign of the association among the feature with the treatment group, with red and green indicating a decrease or increase in the FOOT vs. VOO, FOO vs. VOO, or FOOT vs. FOO, respectively. Significant features were identified using 5000 trees, 500 iterations, and *P*_Bonferroni_ < 0.005. DMA, dimethylamine; DMSO2, dimethylsufone; FOO, functional OO enriched with its own phenolic compounds; FOOT, functional OO enriched with its own phenolic compounds plus additional complementary one from thyme; VOO, virgin olive oil; 3-HB, 3-hydroxybutyrate.

The OPLS-DA results were further validated by ML analyses (Figure 2). Glutamine, creatine, creatinine, DMA, and histidine were consistently decreased after treatment with either FOOT (Figure 2a–c) or FOO (Figure 2d–f) compared to VOO. In addition, FOOT decreased the plasma levels of glycine and DMSO2 compared to VOO (Figure 2c), while FOO also decreased the circulating alanine concentrations but increased the plasma levels of acetone and 3-HB compared to VOO (Figure 2f). Glutamine, creatinine and histidine were the most important metabolites differentiating between the treatments with either FOOT or FOO and VOO. Finally, ML models identified three relevant metabolites discriminative of the treatment with the functional OOs (Figure 2g–i). The circulating levels of isoleucine were increased in the FOOT compared to the FOO, whereas the levels of acetoacetate and 3-HB were increased in the FOO compared to the FOOT.

### 3.3. Metabolic Associations with Cardiovascular Parameters

**Figure 3:**
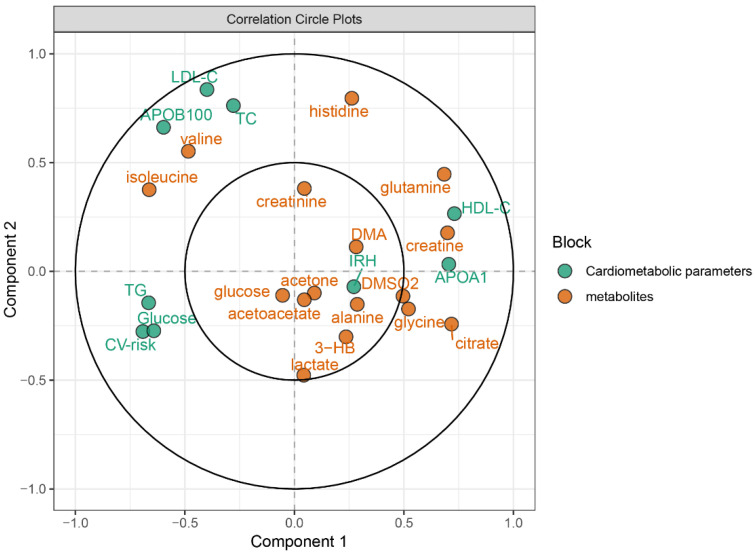
PLS representing the associations between metabolites and cardiometabolic parameters (X1 = metabolites, X2 = cardiometabolic parameters). APO, apolipoprotein; DMA, dimethylamine; DMSO2, dimethylsufone; 3-HB, 3-hydroxybutyrate; IRH: endothelial function; TC, total cholesterol; TG, tryglicerides.

The branched chain amino acids (BCAAs) were moderate positively correlated with APOB100 (isoleucine) and triglycerides (isoleucine), and moderate negatively correlated with HDL-C (isoleucine) and APOA1 (valine and isoleucine) (*p* < 0.002). A number of non-branched chain amino acids were strong negatively correlated with APOB100 (glutamine), glucose (glutamine, histidine), triglycerides (glutamine, histidine), cardiovascular risk (glutamine, histidine), strong positively correlated with HDL-C (glutamine), and APOA1 (glutamine) (*p* < 0.002). In addition, a moderate postitive correlation between APOA1 and glycine was observed (*p* < 0.002) (Table 2 and Figure 3).

## 4. Discussion

In this work we show that the consumption of an OO enriched with either autologous PCs (FOO) or a combination of autologous plus complementary thyme PCs (FOOT) altered the circulating metabolomes of hypercholesterolemic volunteers compared to the consumption of a standard VOO. In particular, reductions were seen in circulating glutamine, histidine, DMA, creatine, and creatinine after both PC-enriched OOs.

Glutamine has been previously associated with cardiovascular diseases with Shah et al. reporting higher peripheral blood glutamine concentrations in cardiovascular disease patients compared to their healthy equivalents [33]. It has also been reported that glutamine promotes the accumulation of macrophage triglyceride by enhancing the uptake of LDL and VLDL [34] and this could promote atherosclerotic plaque formation. It has been described that glutamine could have a positive role reducing oxidative stress, since glutamine is a precursor of glutathione [35]. Interestingly, the essential amino acid histidine, also has antioxidant roles. This includes proton buffering, metal ion chelation, and scavenging of reactive oxygen and nitrogen species [36]. In this study, reductions in these circulating aminoacids could indicate lower systemic demand due to the enhanced antioxidant content of the PC-enriched OO.

Alanine was found decreased only after the FOO intervention in comparison with VOO. The precursors of glutamine and alanine are the BCAA. The initial site of BCAA catabolism is the skeletal muscle and there is a release of glutamine and alanine to the blood during this process [37]. In this study, we also observed reductions in the circulating BCAAs, valine and isoleucine after both PC-enriched OOs. BCAAs have previously been associated with cardiovascular diseases [38], stroke [39], cancer, type 2 diabetes [40], and metabolic syndrome [41,42]. Specifically, valine has been linked with metabolic risk factors [43], insulin resistance [44], incident type 2 diabetes [40], and future cardiovascular events [33]. Moreover, the BCAAs levels of plasma and tissue have been reported to be increased in breast cancer [45]. In agreement, the circulating BCAAs in this study were positively correlated with cardiovascular risk parameters, such as LDL-c, APOB100, triglycerides, and negatively associated with cardiovascular-protective parameters, including HDL-c and APOA1. Such findings concur with previous studies highlighting the positive relationships between BCAAs and triglycerides and the negative association with HDL-c [46]. Collectively, these results suggest that the consumption of a phenol-enriched OO by hypercholesterolemic individuals could lead to favorable shifts in their circulating BCAA-related metabolic phenotype towards a more cardio-protective one.

Several studies have observed a relationship between ketone bodies and cardiovascular risk as they are involved in diabetic ketoacidosis [47]. Conversely, ketone bodies also have bioenergetic and pleiotropic effects that could induce cardiovascular benefits [47]. In this study, serum acetoacetate was reduced by FOOT and increased by FOO, compared to VOO consumption. Furthermore, FOO consumption resulted in higher amounts of serum 3-HB and acetoacetate, compared to FOOT. These results suggest that FOOT may induce more favorable metabolic modifications than FOO. In contrast, circulating levels of isoleucine, a BCAA, were increased in the FOOT compared to the FOO.

Creatinine is a degradation product from creatine, and is a marker of muscle mass, adverse lipid profiles, and kidney dysfunction. Both creatinine and creatine were decreased in serum after the two phenol-enriched OOs in comparison to VOO. Creatinine has been associated with cardiovascular diseases [48,49] and elevations in serum creatinine have been noted with chronic heart failure, acute myocardial infarction, and chronic ischemic heart disease patients [50]. Moreover, increased serum creatinine is a predictor for all-cause mortality [51].

Interestingly, the microbial metabolite DMA was reduced in the circulation after consumption of both phenol-enriched OOs. DMA arises from the bacterial metabolism of trimethylamine and trimethylamine-N-oxide (TMAO), derived from the microbial breakdown of dietary choline and L-carnitine. TMAO promotes atherosclerosis in animal models and is associated with CVD and adverse cardiac events in humans [52,53,54]. The reduction of DMA observed after both phenol-enriched OOs could be related to anti-atherosclerotic properties extensively described in cellular, animal, and human studies.

The similar metabolomic effects observed after both phenol-enriched olive oils intake suggest that the complementarity of the antioxidants does not produce additional beneficial effects in the outcome analyzed in this paper, the metabolome. Nevertheless, our group reported before that this functional olive oil enriched with complementary antioxidants showed a capacity to improve HDL-subclass distribution and composition, and metabolism/antioxidant enzyme activities versus VOO, and the functional olive oil enriched with only its own antioxidants did not show this capacity [55]. In this sense, our group also reported before that FVOOT improved the expression of cholesterol efflux related genes [14].

One strength of our nutritional intervention trial is its randomized and cross-over design, which permitted the volunteers to consume all OO types and therefore deleted the inter-individual variability. In addition, the laboratory analyses were centralized and all the time-series samples from the same participant were measured in the same run. A limitation of the study was its sample size, which could be responsible for reduced statistical power in some metabolites with high inter-individual variability. A synergistic effect on serum metabolome between PC and other OO components is as yet unknown. Another limitation is the inability to assess potential interactions among the OOs and other diet components and medication, although the controlled diet and medication followed during all clinical trial should have limited the aim of these interactions.

In conclusion, dietary supplementation of a regular daily dose (25 mL/day) of phenol-enriched OOs for three weeks reduced several serum metabolites which were associated with cardiovascular-risk, in hypercholesterolemic patients. In particular, two different phenol-enriched OOs reduce the levels of glutamine, histidine, DMA, creatine, and creatinine. Such results highlight the potential of this cost-effective nutritional modification to reduce the global burden of cardiovascular diseases, and lead to its development as a nutritional tool for the treatment of cardiometabolic diseases.

## Figures and Tables

**Table 1:** Baseline characteristics of the participants.

	Order 1 (*n* = 11)	Order 2 (*n* = 11)	Order 3 (*n* = 11)	*p*-Value
**Anthropometric and Cardiovascular Clinical Measurements**				
Sex: man	5 (45.6%)	7 (63.6%)	7 (63.6%)	0.742
Age (years)	54.91 ± 12.57	55.27 ± 11.88	55.45 ± 7.84	0.856
BMI (Kg/m^2^)	25.63 ± 3.68	26.31 ± 5.25	27.85 ± 4.71	0.529
Physical activity (Kcal/week)	3498.75 (1755.00; 8092.50)	1188.75 (742.50; 1687.50)	3322.50 (861.25; 3663.75)	0.094
Ischemic reactive hyperemia (IRH)	268.95 ± 344.05	60.374 ± 74.63	177.51 ± 174.01	0.159
Cardiovascular risk	4.472 ± 2.424	5.525 ± 2.693	4.970 ± 2.124	0.601
**Systemic Lipid Profile and Glycaemia**				
Total cholesterol (mg/dL)	228 ± 43	232 ± 33	219 ± 31	0.680
Triglycerides (mg/dL)	94 (75; 149)	119 (95; 168)	117 (81; 126)	0.517
Glucose (mg/dL)	89 ± 12	93 ± 13	91 ± 11	0.683
HDL-cholesterol (mg/dL)	53 ± 13	53 ± 13	53 ± 20	0.992
LDL-cholesterol (mg/dL)	150 ± 32	152 ± 28	142 ± 26	0.700
Apolipoprotein-A1 (g/L)	1.4 ± 0.2	1.4 ± 0.2	1.5 ± 0.2	0.458
Apolipoprotein-B100 (g/L)	1.2 ± 0.2	1.2 ± 0.2	1.1 ± 0.2	0.529

Values expressed as mean ± S.D. or median (25th to 75th percentile).

**Table 2:** Correlations between ^1^H-NMR significant metabolites and cardiometabolic parameters.

	Isoleucine	Valine (1.03 ppm)	Glutamine	Alanine	Histidine (7.04129 ppm)	Glycine	DMSO_2_	Glucose (4.62975 ppm)	Citrate (2.66236 ppm)	Lactate (1.33017 ppm)	DMA	Creatine	Creatinine	Acetone	Acetoacetate	3-HB
**HDL-C**	*r* = −0.271 *p* = 0.000	NS	*r* = 0.477 *p* = 0.000	NS	NS	NS	NS	*r* = −0.285 *p* = 0.000	*r* = 0.275 *p* = 0.000	NS	NS	*r* = 0.413 *p* = 0.000	NS	NS	NS	NS
**ApoA1**	*r* = −0.303 *p* = 0.000	*r* = −0.298 *p* = 0.000	*r* = 0.356 *p* = 0.000	NS	NS	*r* = 0.246 *p* = 0.001	NS	*r* = −0.248 *p* = 0.001	*r* = 0.242 *p* = 0.001	NS	NS	*r* = 0.318 *p* = 0.000	NS	NS	NS	NS
**Endothelial function**	NS	NS	NS	NS	NS	NS	NS	NS	NS	NS	NS	NS	NS	NS	NS	NS
**Total cholesterol**	NS	NS	NS	NS	NS	NS	NS	NS	NS	NS	NS	NS	NS	NS	NS	NS
**LDL-C**	NS	NS	NS	NS	NS	NS	NS	NS	NS	NS	NS	NS	NS	NS	NS	NS
**ApoB100**	*r* = 0.240 *p* = 0.001	NS	*r* = −0.304 *p* = 0.000	NS	NS	NS	NS	NS	*r* = −0.283 *p* = 0.000	NS	NS	NS	NS	NS	NS	NS
**Triglycerides**	*r* = 0.278 *p* = 0.000	NS	*r* = −0.545 *p* = 0.000	NS	*r* = −0.332 *p* = 0.000	NS	*r* = −0.304 *p* = 0.000	NS	*r* = −0.247 *p* = 0.001	NS	NS	*r* = −0.406 *p* = 0.000	NS	NS	NS	NS
**Cardiovascular risk**	NS	NS	*r* = −0.396 *p* = 0.000	NS	*r* = −0.375 *p* = 0.000	NS	NS	*r* = 0.236 *p* = 0.0017	NS	NS	NS	NS	NS	NS	NS	NS
**Glucose**	NS	NS	*r* = −0.438 *p* = 0.000	NS	*r* = −0.332 *p* = 0.000	NS	NS	*r* = 0.404 *p* = 0.000	NS	NS	NS	*r* = −0.301 *p* = 0.000	NS	NS	NS	NS

Apo, apolipoprotein; DMA, dimethylamine; DMSO2, dimethylsufone; 3-HB, 3-hydroxybutyrate; NS: non-significant.

## Data Availability

The data presented in this study are available in the article and supplementary material.

## Supplementary Materials

The following supporting information can be downloaded at: https://www.mdpi.com/article/10.3390/antiox11101964/s1, Table S1: Chemical characterization of the OOs used in the VOHF study.
