# Supplementary material for: Impact of Phenol-Enriched Olive Oils on Serum Metabonome and Its Relationship with Cardiometabolic Parameters: A Randomized, Double-Blind, Cross-Over, Controlled Trial
Source: Antioxidants (Basel). 2022 Sep 30;11(10):1964. doi: 10.3390/antiox11101964 (PMC9598678; doi:10.3390/antiox11101964)
Supplement: Supplementary file 1 [file antioxidants-11-01964-s001.zip › Supplementary Table 1_MFarr¿ñs_v2.pdf]

Supplementary Table 1. Chemical characterization of the virgin olive oils used in the VOHF study. Phenolic compounds, fat soluble micronutrients and fatty acids daily intake through 25 mL of VOO, FOO and FOOT.

	WASH-OUT OO	VOO	FOO	FOOT
**PHENOLIC COMPOUNDS (mg/25 mL/day)**				
Hydroxytyrosol	0.04 ± 0.00	0.01 ± 0.00	0.21 ± 0.02	0.12 ± 0,00
3,4-DHPEA-AC	n.d.	n.d.	0.84 ± 0.06	0.39 ± 0.04
3,4-DHPEA-EDA*	0.04 ± 0.00	0.04 ± 0.00	6.73 ± 0.37	3.43 ± 0.29
3,4-DHPEA-EA*	0.11 ± 0.00	0.26 ± 0.04	0.71 ± 0.06	0.36 ± 0.03
Total hydroxytyrosol derivates	***0.18***	***0.3***	***8.49***	***4.3***
p-hydroxybenzoic acid	n.d.	n.d.	0.02 ± 0.00	0.06 ± 0,00
Vanillic acid	n.d.	n.d.	0.07 ± 0.00	0.13 ± 0.01
Caffeic acid	n.d.	n.d.	0.00 ± 0.00	0.06 ± 0.00
Rosmarinic acid	n.d.	n.d.	n.d.	0.41 ± 0.03
Total phenolic acids	***-***	***-***	***0.09***	***0.65***
Thymol	n.d.	n.d.	n.d.	0.64 ± 0.05
Carvacrol	n.d.	n.d.	n.d.	0.23 ± 0.02
Total monoterpenes	***-***	***-***	***-***	***0.86***
Luteolin	n.d.	0.04 ± 0.00	0.18 ± 0.02	0.21 ± 0.02
Apigenin	n.d.	0.02 ± 0.00	0.06 ± 0.00	0.1 ± 0.00
Naringenin	n.d.	n.d.	n.d.	0.2 ± 0.02
Eriodictyol	n.d.	n.d.	n.d.	0.17 ± 0.01
Thymusin	n.d.	n.d.	n.d.	1.22 ± 0.09
Xanthomicrol	n.d.	n.d.	n.d.	0.53 ± 0.06
7-methylsudachitin	n.d.	n.d.	n.d.	0.53 ± 0.09
Total flavonoids	***n.d.***	***0.06***	***0.23***	***2.95***
Pinoresinol	n.d.	0.05 ± 0.00	0.12 ± 0.00	0.1 ± 0.05
Acetoxipinoresinol**	n.d.	2.47 ± 0.19	3.66 ± 0.31	3.24 ± 0.28
Total lignans	***n.d.***	***2.52***	***3.78***	***3.34***
**FAT SOLUBLE MICRONUTRIENTS (mg/25 mL/day)**				
α-tocopherol	1.82 ± 0.02	3.27 ± 0.01	3.4 ± 0.02	3.44 ± 0.01
lutein	0.02 ± 0.00	0.05 ± 0.00	0.06 ± 0.00	0.07 ± 0.00
β-cryptoxanthin	0.01 ± 0.00	0.02 ± 0.00	0.03 ± 0.00	0.02 ± 0.00
β-carotene	n.d.	0.01 ± 0.00	0.02 ± 0.00	0.02 ± 0.00
**FATTY ACIDS (relative area %)**				
Palmitic acid (C16:0)	11.96	11.21	11.2	11.21
Stearic acid (C18:0)	3.08	1.92	1.92	1.92
Araquidic acid (C20:0)	0.41	0.36	0.36	0.36
Behenic acid (C22:0)	0.11	0.11	0.11	0.11
Total saturated	***15,67***	***13.75***	***13.74***	***13.75***
Palmitoleic acid (C9-C16:1)	1.00	0.7	0.7	0.69
Oleic acid (C9-C18:1)	72.71	76.74	76.83	76.75
Gadoleic acid (C11-C20:1)	0.25	0.27	0.27	0.27
Total monounsaturated	***73.96***	***77.71***	***77.8***	***77.72***
Linoleic acid	9.39	7.43	7.36	7.43
Timnodonic acid	0.20	0.36	0.36	0.35
Linolenic acid	0.54	0.43	0.43	0.43
Total polyunsaturated	***10.13***	***8.22***	***8.15***	***8.22***

Values are expressed as mean ± standard deviation (SD). The acidic composition is expressed as relative area percentage. VOO, virgin olive oil; FVOO, functional VOO enriched with its own phenolic compounds; FVOOT, functional VOO enriched with its own phenolic compounds plus additional complementary ones from thyme; 3,4-DHPEA-AC, 4-(acetoxyethyl)-1,2-dihydroxybenzene; 3,4-

DHPEA-EDA, dialdehyde form of elenolic acid linked to hydroxytyrosol; 3,4-DHPEA-EA, oleuropeinaglycone; n.d., not detected.
*Quantified with a calibration curve of hydroxytyrosol. **Quantified with a calibration curve of pinoresinol.
